# The times they are AI-changing: AI-powered advances in the application of extracellular vesicles to liquid biopsy in breast cancer

**DOI:** 10.20517/evcna.2024.51

**Published:** 2025-02-28

**Authors:** Vanesa García-Barberán, María Elena Gómez Del Pulgar, Heidy M. Guamán, Alberto Benito-Martin

**Affiliations:** ^1^Molecular Oncology Laboratory, Medical Oncology Department, Hospital Clínico Universitario San Carlos, Instituto de Investigación Sanitaria San Carlos (IdISSC), Madrid 28040, Spain.; ^2^Facultad de Medicina, Universidad Alfonso X el Sabio, Madrid 28691, Spain.

**Keywords:** Extracellular vesicles, breast cancer, artificial intelligence, liquid biopsy

## Abstract

Artificial intelligence (AI) is revolutionizing scientific research by facilitating a paradigm shift in data analysis and discovery. This transformation is characterized by a fundamental change in scientific methods and concepts due to AI’s ability to process vast datasets with unprecedented speed and accuracy. In breast cancer research, AI aids in early detection, prognosis, and personalized treatment strategies. Liquid biopsy, a noninvasive tool for detecting circulating tumor traits, could ideally benefit from AI’s analytical capabilities, enhancing the detection of minimal residual disease and improving treatment monitoring. Extracellular vesicles (EVs), which are key elements in cell communication and cancer progression, could be analyzed with AI to identify disease-specific biomarkers. AI combined with EV analysis promises an enhancement in diagnosis precision, aiding in early detection and treatment monitoring. Studies show that AI can differentiate cancer types and predict drug efficacy, exemplifying its potential in personalized medicine. Overall, the integration of AI in biomedical research and clinical practice promises significant changes and advancements in diagnostics, personalized medicine-based approaches, and our understanding of complex diseases like cancer.

## ARTIFICIAL INTELLIGENCE: THE OLD ROAD IS RAPIDLY AGING

The application of artificial intelligence (AI) in science is triggering a real-time paradigm shift in how research and discovery are conducted. A paradigm shift in science philosophy, a term coined by Thomas Kuhn in “The Structure of Scientific Revolutions”^[1]^, refers to a “fundamental change in the basic concepts and experimental practices of a scientific discipline”. Paradigm shifts occur when the accumulation of anomalies - observations or problems that cannot be explained by the current paradigm - reaches a critical point. Such shifts are revolutionary rather than evolutionary, leading to a profound rethinking of scientific concepts and methods. As of 2024, AI enhances data analysis by processing vast datasets with unprecedented speed and accuracy, uncovering patterns and insights otherwise invisible^[2,3]^. This capacity of AI to reveal novel, previously undetected scientific pathways is where the potential for a paradigm shift lies^[4]^. Its ramifications are growing every day. AI is the general concept for the establishment of intelligent computational agents that carry out tasks demanding human-level intelligence^[5]^. There are different subsets of AI. In particular, machine learning (ML) focuses on algorithms that make systems improve themselves from output. ML algorithms, such as decision trees or support vector machines (SVMs), learn from data and make predictions or decisions^[6]^. While ML encompasses a wide range of techniques for teaching computers to learn from data, deep learning (DL) is a specialized subset that utilizes artificial neural networks with multiple layers to model complex patterns and search for concise outputs^[7]^. ML algorithms predict outcomes and optimize experimental conditions. AI-driven simulations and models enable complex systems exploration and facilitate interdisciplinary collaboration by integrating diverse data sources and methodologies. Data preparation is a very significant stage in ML, which includes cleaning, transformation, and organization of data to make it appropriate for model training^[8]^. It involves handling missing values, outliers, and noise (irrelevant or inaccurate information that could negatively impact a model’s performance); feature creation and transformation; normalization or standardization of data; class imbalance; and splitting data into training and testing. Once the data are prepared, model selection becomes crucial. This involves comparing different algorithms and tuning their hyperparameters using techniques like grid search and cross-validation. Grid search systematically explores different combinations of hyperparameters, while cross-validation helps to assess a model’s generalization performance. By carefully selecting the appropriate model and tuning its parameters, one can achieve optimal performance on unseen data.

Medical practice will not be an exception to the AI revolution. AI is enhancing diagnostic accuracy, treatment personalization, and healthcare efficiency^[9]^. AI-driven algorithms can analyze vast amounts of medical data to identify patterns and predict disease outcomes with greater precision than traditional methods. AI will soon be capable of designing personalized therapies, including drug repurposing, a promise that has been in development for decades. The new scenario connecting AI and biomedicine is a blooming field with new and exciting applications. Recent advances in predicting structure-activity relationships^[10]^, designing self-assembly nanoparticles^[11]^, or highly accurately predicting protein structure^[12,13]^ are transforming our expectations on how computer science can be applied to biomedicine.

## DL: ML’S NEXT EVOLUTION

ML transforms the inputs of an algorithm into outputs by using statistical, data-driven rules that are automatically inferred from a large set of examples, rather than being specified by humans^[7]^. DL is a form of representation learning, a ML technique that automatically discovers meaningful patterns, in which the input is raw data, and it is able to develop its own representations needed for pattern recognition^[14]^. The current revolution associated with the improved performance of DL vs a broader ML approach relies on DL’s capacity to accept multiple data types as input. Many biomedical datasets are composed of input data points (e.g., skin lesion images) and corresponding output data labels (e.g., “benign” or “malignant”). Neural networks, particularly DL, excel at processing complex biomedical data, such as genomics and proteomics, enabling the discovery of new biomarkers and therapeutic targets^[15]^. We can classify DL architectures into four groups: convolutional neural networks (CNNs), recurrent neural networks (RNNs), deep neural networks (DNNs), and emergent architectures.

CNNs are a type of neural network designed to process visual data, making them ideal for tasks like image and video recognition. CNNs are architectures particularly capable of processing image recognition tasks and consist of convolution layers, non-linear layers, and pooling layers^[16]^. RNNs are neural networks that are well-suited for processing sequential data, such as time series data. RNNs are designed to use sequential information of input data with cyclic connections among building blocks^[17]^. DNNs are renowned for their suitability in analyzing high-dimensional data. Their potential covers hierarchical representation learning methods, and could discover previously unknown patterns and correlations, providing a revolutionary way of looking at the data. However, their capabilities are not fully exploited due to a lack of data standardization and persistent challenges that hinder AI applications. One of the earliest DNN applications was on breast imaging, circa 1996^[18]^. Unfortunately, large public digital databases, crucial for algorithm training, are unavailable, further contributing to slow advances.

Diverse datasets require distinct approaches to achieve expected results. As mentioned, image data are best handled by CNNs due to their ability to recognize spatial hierarchies, while sequential data like time series or text are better processed by RNNs^[9]^. Some AI techniques, such as DL, require substantial computational power and large datasets, whereas others, like SVMs or k-nearest neighbors (KNN), can be effective with fewer data and lower computational requirements^[19]^. Other methods, like reinforcement learning, are particularly useful in scenarios involving decision making, whereas clustering algorithms are essential for segmenting datasets into meaningful groups. ML algorithms analyze datasets, identifying patterns and predicting outcomes, which is crucial for disease diagnosis and treatment planning.

## BIOMEDICINE IN AI TIMES: DO NOT CRITICIZE WHAT YOU CANNOT UNDERSTAND

AI technologies are increasingly transforming the field of biomedicine, offering a range of benefits that enhance both healthcare delivery and patient outcomes. The advancements offer many opportunities for healthcare professionals and patients alike^[20]^. DL-related methods have become AI dominant applications in AI-based diagnosis and are used in multiple tasks, such as disease classification^[21,22]^, Region Of Interest segmentation^[23]^, medical object or specific cell subtype detection^[24,25]^, and image registration^[26]^. DL applications to predict breast cancer (BC) from mammograms are leading to AI implementation in cancer diagnosis. McKinney *et al.* (2020) evaluated how good DL models could get, complementing mammography-based diagnosis^[27]^. They found that modeling could correctly identify many undiagnosed cancers. Even more, DL is able to classify histopathological images in BC^[28]^ carcinoma and non-carcinoma with a 97.73% sensitivity for carcinoma classification, with an overall accuracy of 95.29%^[29]^. However, new ML avenues are yet to be explored. A combination of ML with an AdaBoost algorithm presents higher sensitivity (98.3%) and accuracy (97.2%), reporting 96.5% specificity and increased patient survival rate^[30]^. Boosting is a general ensemble method that creates a strong classifier built from several weak ones with an increased prediction capacity and minimized error. Genome-wide association studies demand algorithms due to large patient cohorts and confounders, many times unknown. Stochastic optimization of algorithms^[31]^ adapted for DL provides new insights that, combined with other bioinformatics tools, would identify disease-associated causal mutations and help clarify confounder influence^[32]^. Determination of pathogenic variants in genetic diagnosis also benefits from DL, as the prediction of protein structure has transformed medical biochemistry^[33,12]^. DL systems could enhance targeted biomarker assays, for example, for gene expression profiles^[12]^. However, it is the combination of the previously described AI tools that will further advance its applicability. Functional enrichment analysis and Gene Set Enrichment Analysis are also AI techniques. They can analyze if a certain protein or gene expression contributes to statistically significant alterations in their expression, regulation pattern, or specific biological function. These are enrichment score analyses and rely on known biological pathways from existing databases such as the Kyoto Encyclopedia of Genes and Genomes (KEGG)^[34]^ or Gene Ontological Resource (GO)^[35]^. Once DL organizes data patterns, functional enrichment tools map them to known biological pathways and functions. This integration allows researchers to gain deeper insights into the underlying biological mechanisms and potential associations with diseases. By bridging advanced data analysis with biological context, this approach enhances our understanding of complex biological systems and supports the discovery of novel therapeutic targets or biomarkers in biomedicine. Complemented with AI machinery, enrichment analysis provides a new direction to integrative “multiomics”.

**Figure 1:**
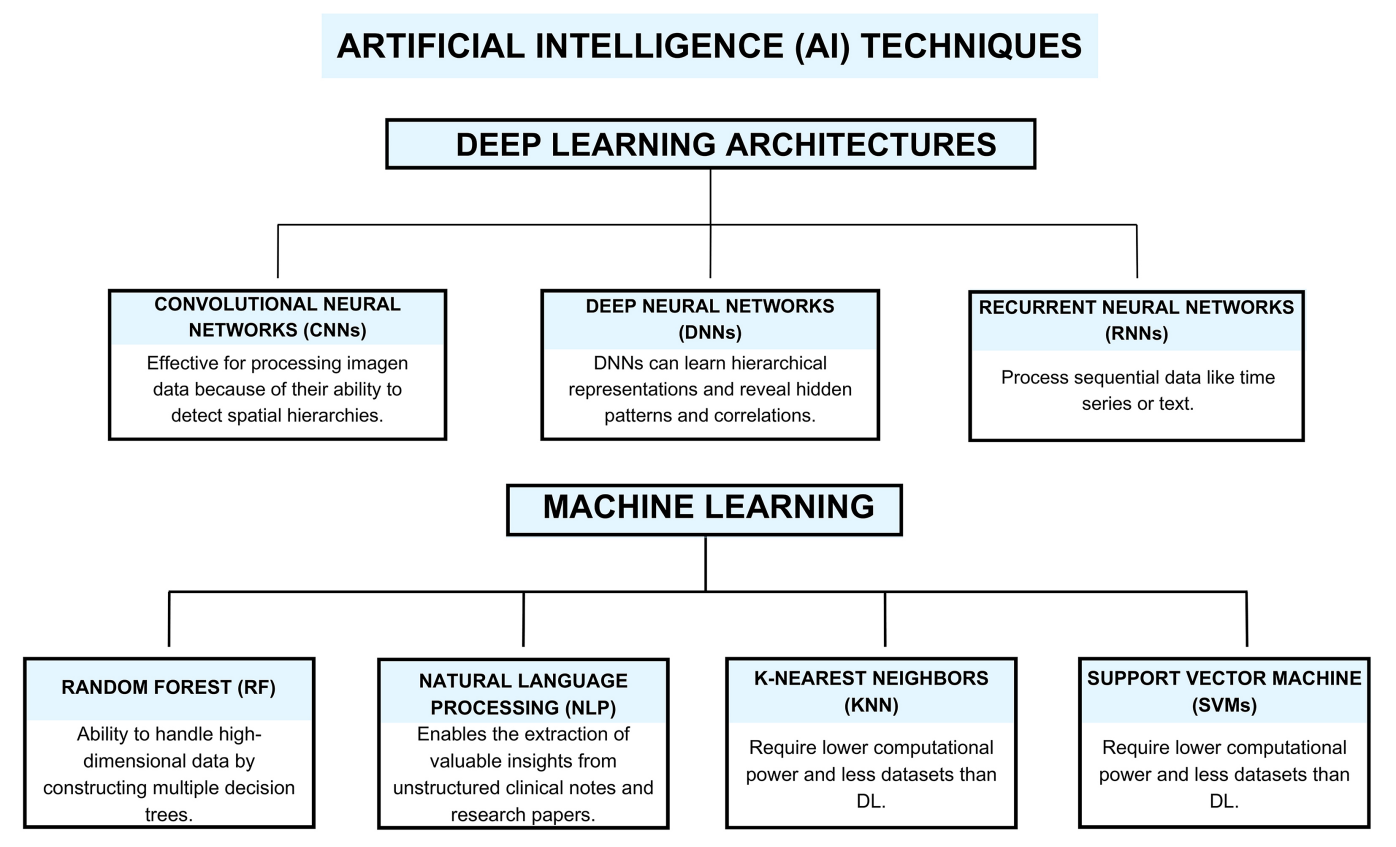
Schematic representation of the different AI strategies applied to biomedical research.

Supervised learning models are trained on labeled medical images to detect abnormalities like tumors with high accuracy^[36]^. Other applications include natural language processing (NLP), which facilitates the extraction of valuable insights from unstructured clinical notes and research papers. Random forest (RF), created by Leo Breinman in 2001^[37]^, is an ensemble learning method that combines the predictions of multiple decision trees, each trained on a different data subset and considering a random selection of features. The RF strategy enhances the accuracy and robustness of the model by reducing overfitting through the combination of multiple trees. RF is widely applied in biomedicine for its robustness and ability to handle high-dimensional data^[38]^. It is used in disease diagnosis and classification, helping to identify diseases such as cancer^[39]^, diabetes^[40]^, and cardiovascular disease^[41]^ based on clinical and genomic data. RF also assists in biomarker discovery by identifying important variables from large datasets, enhancing personalized medicine^[42]^. Its versatility and accuracy make RF a valuable tool in biomedical research and clinical practice. Together, these AI techniques, summarized in Figure 1, are revolutionizing biomedicine by advancing diagnostics, personalized medicine, and biomedical research.

## BC AND LIQUID BIOPSY

BC is one of the most common cancers in women, with 2.3 million new cases diagnosed globally in 2022^[43]^. BC shows different forms of presentation. Roughly 70% of all cases of BC are classified as sporadic, 20% as familial BC, and 10% as hereditary BC. A large proportion of BCs occur in a small population percentage, who are at increased risk of developing the disease^[44]^. Susceptibility to BC is multifactorial and many genetic variants and reproductive, hormonal, anthropomorphic, and lifestyle factors are considered associated risk factors. Each of these factors might have a modest effect on cancer risk, but when considered together with family history and known genetic factors, they can improve patient risk stratification. In a large study evaluating variation in putative BC susceptibility genes, authors found strong evidence of an association with BC for protein-truncating variants in 9 genes, including *ATM*, *BRCA1*, *BRCA2*, *CHEK2*, and others^[45]^. Patients carrying these mutations are considered at high risk of developing hereditary BC.

BC is a heterogeneous disease classified into three major intrinsic subtypes^[46]^ based on immune-histochemical expression of hormone receptors (estrogen/progesterone receptor positive), and human epidermal growth factor receptor amplification (HER2). Hormone receptor-positive (luminal) BC accounts for 65% of tumors, and they show a low rate of distant relapse. Nearly 15% of all the tumors are classified as triple-negative breast cancer (TNBC), which lacks the expression of HER2 and hormone receptors, and 15-20% are HER2+ (non-luminal) subtype^[47]^, both of which are associated with a worse prognosis. In addition to the outcome, treatment is determined by subtype. Thus, therapies are mainly based on endocrine therapy for luminal tumors, HER2 inhibitors for HER2+ tumors, and chemotherapy (CT) for TNBC. Despite the improvement in early detection and therapies, a group of patients will relapse over the years. Prognostic and predictive factors are useful tools to predict relapse risk and individualize anticancer drug therapies. The integration and AI shows promise in early diagnosis and in identifying patients at high risk of relapse during adjuvant and neoadjuvant treatments (NAT)^[48]^. Although adjuvant hormone therapy is the standard of care in early Luminal A BC, a subset of high-risk patients could also benefit from additional adjuvant CT for the prevention of recurrence. However, CT toxicity has a negative impact on the quality of life of these patients. Therefore, identifying these patients is essential to deciding the best therapeutic strategy, sparing unnecessary over-treatment and CT toxicity in patients with low risk, while increasing the lifesaving potential of CT treatment in high-risk patients. In order to accurately identify the genomic risk, several multigene expression profile platforms have been developed, such as Oncotype DX, Prosigna, or MammaPrint^[49]^. Oncotype DX is also a predictive test that provides information on the benefits of CT in adjuvant treatment^[50]^. Despite efforts to improve the adjuvant treatment management of these patients, between 25% and 40% of them will present a locoregional or distant relapse. In the same way, the benefit of NAT in locally advanced diseases varies based on the individual risk of relapse. HER2+ and TNBC subtypes show high sensitivity from NAT. Two out of three patients achieve pathological complete response (pCR) in TNBC, while only nearly half of patients in HER2+ BC^[47]^. Achieving pCR after NAT identifies patients with a lower risk of relapse, as indicated by improved relapse-free survival, both in HER2+ and TNBC^[51]^. However, non-pCR patients are at a higher risk of experiencing cancer recurrences, and these cohorts should be treated with additional adjuvant therapies. New strategies to predict and stratify patients based on their prognosis are highly demanded by patients and clinicians.

Liquid biopsy is a noninvasive diagnosis tool that detects circulating tumor components, including circulating tumor cells, circulating tumor DNA (ctDNA), circulating miRNAs, extracellular vesicles (EVs) and particles, soluble proteins, mRNAs, and other elements present in patients’ peripheral blood or other biological fluids. This biopsy strategy provides a comprehensive view of the disease status, enabling continuing analysis and aiding clinical decision making throughout specific therapeutic approaches^[52]^. Monitoring any dynamic alterations in liquid biopsy during treatment may report efficacy information to facilitate personalized medicine. Tumor mutations in ctDNA are being used to identify minimal residual disease in different types of cancer, including BC, with promising results^[53,54,55]^. So far, only a few studies based on deep sequencing and ctDNA dynamics have addressed liquid biopsy efficacy in patients receiving NAT for TNBC and HER2+ subtypes. Butler *et al.* analyzed ctDNA in three TNBC and three HER2+ patients, detecting ctDNA prior to the start of NAT based only on CT, decreasing during treatment in patients with pCR and increasing in rapid recurrences^[56]^. McDonald *et al.* demonstrated a decrease in ctDNA concentrations in patients with pCR during NAT, but only 7 and 9 patients with HER2+ and TNBC, respectively, were included in the series^[57]^. Similarly, Li *et al*. observed an association between response to NAT and ctDNA detection after two cycles in 44 BC patients (of whom 6 were TNBC and 9 HER2+) by Li and colleagues^[58]^. Magbanua *et al.* evaluated ctDNA in neoadjuvant-treated high-risk early BC patients (MammaPrint high score) included in the I-SPY 2 trial^[59]^. Eighty-four patients treated with a standard NAT alone or in combination with an AKT inhibitor were analyzed using an NGS panel (16 highly ranked somatic mutations). Probably, analysis of one alteration per patient impairs the real percentage of detection of molecular disease in liquid biopsy, making room for novel strategies.

This is where AI enters the stage. The scientific community is taking multiple approaches to integrate the potential of AI applications into BC research. Combined strategies aim to distinguish healthy and cancer cells^[60]^. Multi-modal and “-omics” ML integration aims to enhance drug-response prediction in BC patients, distinguishing non-responders and variable responders^[61]^. Some strategies try to improve the prediction accuracy of magnetic resonance imaging^[62]^ or tissue morphology correlations^[63]^. Retrospective studies to expand the validity of mammograms could improve diagnosis and include accurate predictive risk^[64,65]^. Other applications aim to address tumor evolution based on oscillating gene expression^[66]^, improve tumor subclassification^[67]^, or integrate genotype-phenotype association^[68]^. ML has also been applied to prediction models for the impact of CT. In a cohort of 4,696 patients, the propensity-score-matched method was utilized to reduce covariable imbalance. Univariable and multivariable analyses were used to compare BC-specific survival and overall survival^[69]^.

## EVs AND AI

EVs are key factors for cell-to-cell communication, playing a role in metastatic dissemination and cancer progression^[70,71]^. These vesicles, released from almost all cell types and organisms studied, bear a resemblance to their cell of origin in terms of their protein, lipid, and nucleic acid content^[70]^. Several works have reported that EV-shed DNA allows the detection of mutations that reliably reflect the mutational state in the tumor of origin^[72,73,74]^. Even more, EV-associated DNA shows higher accordance with conventional tissue biopsy compared to the liquid biopsy of cfDNA^[75]^. According to their physical functions, EVs have been used in therapeutic agents, vaccination trials, regenerative medicine, and drug delivery^[76,77,78]^. The application of knowledge about EVs in liquid biopsy strategies spans various biological fluids^[79]^. For example, serum, plasma, and cerebrospinal fluid were EV-DNA sources for predictive detection of BRAF mutations in pediatric central nervous system tumors^[80]^. BRAF mutation was also the target of post-lymphadenectomy seroma analysis of EV-DNA obtained from melanoma patients^[81]^. The results from this study indicate that EV-derived DNA from seroma fluid may provide a promising tool for the detection of minimal residual disease in BC and melanoma, where lymph node removal is frequently performed. Moreover, the investigation into circulating EV-DNA in conjunction with other factors may provide a prognosis for various types of cancer. The fact that EVs contain a diverse array of contents suggests that the development of assays that analyze DNA alongside other biomolecules may enable personalized treatments for cancer. Traditionally, the validity of EVs as biomarkers has been hampered by sample and patient heterogeneity. A typical EV proteomic data set generally includes thousands of protein identifications, with low uniqueness. Therefore, the lack of appropriate tools to analyze and correlate their content with other parameters has limited EV application to clinical practice. The combination of AI and the study of EVs is on the rise as it could fill that gap. As we have discussed above, the multiple strategies encompassed by the general term AI engulf a variety of exciting new roads. Recently, Greenberg *et al.* reviewed EVs’ role in emerging drug delivery approaches and how AI could contribute to the field expansion^[82]^. The authors actively recommend following International Society for Extracellular Vesicles (ISEV) guidelines(MISEV)^[83]^ to improve standardization and decrease the confusing information associated with particle heterogeneity. MISEV guidelines briefly discuss AI, but the society’s effort to standardize EV research would foster EV-related data applicability to AI research. AI application to basic research is in its early stages, and poor model standardization could delay effective implementation. One of the highlights of the novel relationship between AI and EVs was produced by Hoshino *et al.*^[42]^. The authors applied proteomics to investigate tumor EV heterogeneity to define marker targets using RF and principal component analysis. In their study, they standardized and analyzed hundreds of EV proteomic data sets using ML to identify EV markers and populations corresponding to specific tumor origins. RF implementation could potentially enable faster cancer patient diagnoses when applied to liquid biopsy. Employing datasets of EV-containing proteins from human cell lines, tissue, plasma, serum, and urine samples from a variety of cancers, other groups propose three panels of pan-cancer EVs proteins that distinguish cancer EVs from other vesicles and aid in classifying cancer subtypes employing RF models^[84]^. Using CNN, it is now possible to detect and profile apoptotic events, aiming to overcome the limitations associated with unspecific staining, poor timing in biological process measurements, or inconsistent and late indication of programmed cell death onset^[85]^. Considering the long conflict involving the differentiation of EVs and apoptotic bodies, the application of AI presents promising opportunities. Multi-omics strategies provide complex information that could help to elucidate interactions contributing to pathological states. For example, this approach was used to investigate the heterogeneous and hierarchical organization of lung adenocarcinoma using EVs. Their integrative analysis identified specific roles of RNA-enriched EVs in tumorigenesis, offering a new perspective on liquid biopsy biomarkers for lung adenocarcinoma diagnosis^[86]^. Integration of ML algorithms with DNA cascade reaction-triggered individual EV nanoencapsulation resulted in differential diagnosis accuracy, effectively distinguishing pathological and healthy liver conditions^[87]^. Combinatory strategies like all the previously mentioned illustrate the possibilities for cancer research.

## EVS AND BC DIAGNOSIS WITH AN AI TOUCH

Since their inception, AI algorithms have been paving the way for new approaches to medical diagnosis for oncology. Techniques that range from classification to regression come into play, aided by ML for detecting patterns in medical data, while DL has great performances for medical imaging by CNNs and for time-series data based on RNNs^[88]^. NLP facilitates clinical text analysis with a wealth of useful information in the decision-making process. By leveraging these powerful algorithms, AI empowers oncologists to make more accurate and timely diagnoses, leading to improved patient outcomes. The paradigm shift has already been applied to BC research^[89]^. AI algorithms review a large volume of medical data, including mammograms^[90,91]^ and genetic information, searching for patterns that could predict the disease more accurately^[92]^. This enables earlier detection and more personalized treatment^[93]^. The AI-powered drug discovery accelerates the development of new therapies by screening potential drug candidates at an unprecedented rate. By automating routine tasks and providing informed insight, AI thus enables researchers and clinicians to make better-informed decisions that will bring about real improvements in patient outcomes.

**Figure 2:**
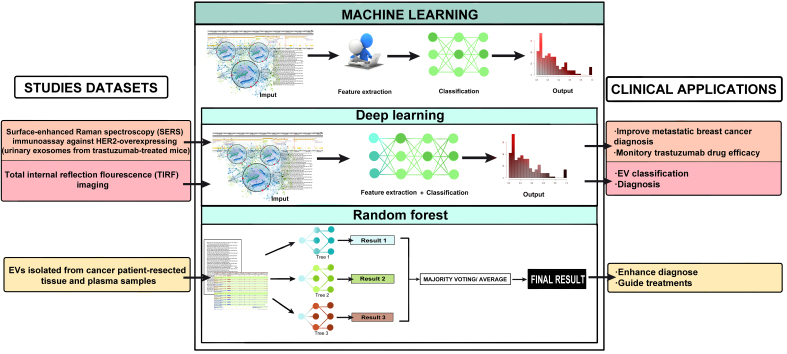
Graphic summary of EV-based liquid biopsy studies applied to breast cancer clinical practice. EV: Extracellular vesicles.

There is potential for large datasets analysis based on EVs that would help to identify patterns and BC biomarkers. This would enhance early detection, prognosis, and personalized treatment strategies by improving the accuracy and efficiency of diagnostic processes. The integration of AI with EV analysis represents a promising frontier in the fight against BC, offering the potential for more precise and noninvasive diagnostic options. Very few studies published in recent years have already combined AI and EVs to improve BC diagnosis, monitoring, or to advance our knowledge of drug efficacy [Figure 2]. However, there are reasonable expectations on how they will impact patient outcomes and prognosis. Total internal reflection fluorescence imaging combined with CNN enables simultaneous and accurate detection of multi-miRNAs at a single EV level. Through the evaluation of three miRNAs using this methodology, Zhang *et al.* confirmed the heterogeneity of EV miRNA expression, revealing that the main variation between EVs from five cancer cells and normal plasma is the triple-positive EV subpopulation^[94]^. The classification accuracy of single triple-positive EVs from six sources can reach above 95%. In the clinical cohort, 20 patients (Breast, lung, cervical and colon cancer, 5 patients each) and five healthy controls are predicted with an overall accuracy of 100%. Using a combination of DL and surface-enhanced Raman spectroscopy (SERS) immunoassay against HER2-overexpressing urinary EVs, some authors are trying to improve treatment efficacy monitoring in metastatic BC. SERS is a technique that significantly amplifies the Raman scattering signal of molecules adsorbed on certain metal surfaces. SERS is widely used in chemical and biological sensing, material science, and environmental monitoring due to its high sensitivity and specificity^[95]^. Drug efficacy was monitored via SERS-DL analysis using urinary EVs from trastuzumab-treated mice^[96]^. Although this is a preclinical application, it clearly illuminates new possible strategies. The combination of label-free EVs SERS and ML could serve as an innovative strategy for medical diagnosis and therapeutic intervention, as some authors are trying to implement it in various scenarios, such as renal injury induced by cisplatin^[97]^. DL algorithm trained with SERS spectra of EVs derived from cancer cells presents high prediction accuracy for human patients with different BC subtypes who do not undergo surgery^[98]^.

Hoshino *et al*. studied characterized EVs isolated from cancer patient-resected tissue and plasma samples including BC samples. They showed that different cancer types, including pancreas, lung, or BC could be distinguished through specific combinations of EV proteins. These cancer-type-specific EV protein signatures could be used as a liquid biopsy tool to help diagnose and guide treatments for these patients. The usage of RF reduced the risk of over-fitting and made the method robust to outliers and noise in the input data^[42]^.

## SUMMARIZING THE FUTURE DIRECTIONS AND DOWNSIDES OF AI APPLICATION TO EV USAGE IN BC LIQUID BIOPSY

There is a bright, exciting and uncertain future for AI in BC research. With enhanced quality and quantity of data, refining DL architectures, and the development of explainable AI, researchers would make AI models more accurate and interpretable. These advances are poised to enable early detection with AI-enhanced imaging^[90]^ and risk prediction, precise diagnosis using digital pathology and molecular subtyping, optimized treatment planning, and better monitoring and prognosis. Future AI research would be oriented toward improving model intelligibility to gain clinical trust, integrating multi-data platforms such as genomics, pathology, and radiology for personalized medicine, and employing federated learning to safeguard data privacy while enabling the use of larger, collaborative datasets. The implementation of AI-driven analysis of EVs in BC diagnosis faces several challenges. First, the collection and isolation of EVs from biological fluids requires standardization to ensure consistency and reliability. Advances in EV isolation, high-throughput technologies, and nanoscale engineering could facilitate the efficient and consistent collection of EV samples. Second, the development and validation of AI algorithms require extensive, high-quality datasets. Integration into clinical practice demands significant investments in infrastructure and training for healthcare professionals: clinicians need to understand and trust these systems before they can be widely adopted. Educational initiatives to train professionals in AI and bioinformatics would help integrate these technologies into clinical practice. However, it is not only a human problem. One major concern is the quality and representativeness of the training data used to generate AI applications, as biased or incomplete datasets can lead to inaccurate and non-generalizable AI models. High-quality datasets are the foundation and a critical point of robust and reliable AI models in medical diagnosis. To ensure the accuracy and generalizability of these models, careful attention must be paid to data collection, annotation, cleaning, and augmentation. Data should be collected from diverse populations to minimize bias and ensure the model’s ability to perform well in different patient groups. Accurate and consistent annotation of data is essential for training effective AI models. Data cleaning techniques, such as handling missing values and outliers, can significantly improve model performance. Data augmentation techniques, such as rotation, flipping, and adding noise, can help address the challenges of small and imbalanced datasets. Additionally, sharing and collaborating on datasets can facilitate the development of more powerful and reliable AI models. By addressing these key considerations, researchers can develop AI models that have the potential to improve patient outcomes. Algorithms being developed and used in health - most of them using patient data - pose critical ethical concerns^[99]^. This is important for compliance with applicable laws and regulations, including but not limited to Health Insurance Portability and Accountability Act (HIPAA) in the US and General Data Protection Regulation (GDPR) in the EU, for maintaining legal and ethical standards. It is crucial to balance data sharing with privacy and ethical considerations to protect patient confidentiality^[100]^. AI models often rely on vast amounts of patient data for training and operation, necessitating compliance with these regulations to ensure the ethical and secure use of sensitive health information. The arcane nature of many AI algorithms poses another challenge, as the lack of transparency in decision making could feed the already detectable trust and acceptance problems among clinicians and patients. As advancements and downsides converge and evolve, AI-driven EV analysis offers a promise to become a routine, noninvasive diagnostic tool that significantly improves early detection, patient outcomes, and personalized treatment strategies in BC treatment. The times, they are changing.

## DECLARATIONS

### Copyright

© The Author(s) 2025.
